# *Lactobacillus acidophilus* inhibits bone loss and increases bone heterogeneity in osteoporotic mice via modulating Treg-Th17 cell balance

**DOI:** 10.1016/j.bonr.2018.02.001

**Published:** 2018-02-05

**Authors:** Hamid Y. Dar, Prashant Shukla, Pradyumna K. Mishra, Rajaneesh Anupam, Rajesh K. Mondal, Geetanjali B. Tomar, Versha Sharma, Rupesh K. Srivastava

**Affiliations:** a Department of Zoology, School of Biological Sciences, Dr. Harisingh Gour Central University, Sagar, MP 470003, India; b Department of Physics, School of Mathematical and Physical Sciences, Dr. Harisingh Gour Central University, Sagar, MP 470003, India; c Department of Molecular Biology, National Institute for Research in Environmental Health, Bhopal, MP 462001, India; d Department of Biotechnology, School of Biological Sciences, Dr. Harisingh Gour Central University, Sagar, MP 470003, India; e Department of Microbiology, School of Biological Sciences, Dr. Harisingh Gour Central University, Sagar, MP 470003, India; f Institute of Bioinformatics & Biotechnology, Savitribai Phule Pune University, Pune, MH 411007, India; g Department of Biotechnology, All India Institute of Medical Sciences (AIIMS), New Delhi, 110029, India

**Keywords:** Probiotics, *Lactobacillus acidophilus*, Bone heterogeneity, Treg cell, Th17 cell, Osteoporosis

## Abstract

Osteoporosis is one of the most important but often neglected bone disease associated with aging and postmenopausal condition leading to bone loss and fragility. Probiotics have been associated with various immunomodulatory properties and have the potential to ameliorate several inflammatory conditions including osteoporosis. *Lactobacillus acidophilus* (LA) was selected as probiotic of choice in our present study due its common availability and established immunomodulatory properties. In the present study, we report for the first time that administration of LA in ovariectomized (ovx) mice enhances both trabecular and cortical bone microarchitecture along with increasing the mineral density and heterogeneity of bones. This effect of LA administration is due to its immunomodulatory effect on host immune system. LA thus skews the Treg-Th17 cell balance by inhibiting osteoclastogenic Th17 cells and promoting anti-osteoclastogenic Treg cells in ovx mice. LA administration also suppressed expression of osteoclastogenic factors (IL-6, IL-17, TNF-α and RANKL) and increased expression of anti-osteoclastogenic factors (IL-10, IFN-γ). Taken together the present study for the first time clearly demonstrates the therapeutic potential of LA as an osteo-protective agent in enhancing bone health (via tweaking Treg-Th17 cell balance) in postmenopausal osteoporosis.

## Highlights

• Post-menopausal osteoporosis is one of most commonly occurring skeletal diseases leading to bone loss and fragility.
• Probiotics possess various immunomodulatory properties and have the potential to ameliorate several inflammatory conditions including post-menopausal osteoporosis.
• We report for the first time that administration of a common probiotic *Lactobacillus acidophilus* (LA) in ovx mice skews Treg-Th17 cell balance in vivo by inhibiting osteoclastogenic Th17 cells and promoting anti-osteoclastogenic Treg cells.
• Also, LA administration suppresses expression of osteoclastogenic (IL-6, IL-17, TNF-α and RANKL) and enhances expression of anti-osteoclastogenic (IL-10, IFN-γ) factors in ovx mice.
• The present study for the first time clearly demonstrates the therapeutic potential of LA as a potent osteo-protective agent in enhancing bone health in post-menopausal osteoporosis.

## Graphical abstract

*Lactobacillus acidophilus* administration bone mass in post-menopausal osteoporotic mice by skewing Treg-Th17 balance.

**Figure:**
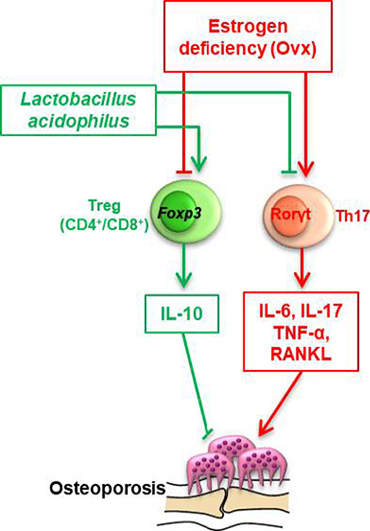

## 1 Introduction

Osteoporosis is an increasingly common chronic condition of bones with >200 million affected individuals worldwide (Laird et al., 2017) Osteoporosis is associated with reduced density and quality of bone leading to weakened skeleton thereby increasing the risk of fractures responsible for increased morbidity and mortality (Haczynski and Jakimiuk, 2001). In addition, osteoporosis will take a heavy toll on the economy with an estimated burden of USD 131.5 billion worldwide by 2050 (Haczynski and Jakimiuk, 2001). Postmenopausal osteoporosis is one of the commonly occurring skeletal diseases promoting bone resorption, enhancing bone loss and fragility in women over 45 years of age (Haczynski and Jakimiuk, 2001). Reduction in ovarian production of estrogen in postmenopausal phase is the main cause for initial phase of rapid bone loss in women, with an annual bone loss rate of 3–5% (Manolagas, 2010). The postmenopausal osteoporosis prevalence and severity are modulated by nutrition, smoking, body mass index, genetic factors and aging (Manolagas, 2010). At the cellular level, the central mechanism by which sex steroid deficiency induces bone loss is via an increase in osteoclast formation and osteoclast lifespan (Manolagas, 2010; D'Amelio et al., 2008). In humans, estrogen deficiency is associated with an expansion of RANKL and TNF-α expressing T cells (Zhao et al., 2016). Estrogen deficiency have been related for setting up of a chronic inflammatory state which in turn leads to bone loss, and this breaking up of the inflammatory cascade at any point can prove to be effective in alleviating bone loss in different animal models (Gao et al., 2007). Despite all the current treatments available for promoting bone health, the number of osteoporotic patients is on the rise worldwide. In addition, conventional bone loss treatments have unwanted side effects and are not always effective. Therefore, new approaches to enhance bone health are needed.

Recently the importance of probiotics as a driver of health had emerged as a novel approach for treating various inflammatory diseases including bone health. Probiotics are live microbial feed supplements that when administered in adequate amounts confer various health benefits to the host (Yousf et al., 2015). Several strains of *Lactobacillus* have been reported with therapeutic effect in experimental models of rheumatoid arthritis (RA), inflammatory bowel disease, atopic dermatitis etc. (Petersen et al., 2012). Various species of *Lactobacillus* such as *L. acidophilus*-NCFM and *L. salivarius* Ls-33 completely protected SCID mice from colitis (Narva et al., 2004). Several strains of *Lactobacillus* are now being employed as effective therapeutics for treatment of various diseases including osteoporosis, as administration of certain probiotics blunts bone loss that usually follows ovariectomy (Chiang and Pan, 2011; Ohlsson et al., 2014; Britton et al., 2014). This approach can be highly invaluable for prevention of increased bone joint destruction in patients where inflammation and bone loss cannot be prevented by established older methods. The mechanism of probiotics effecting bone health had been a topic of research in the last few years, surprisingly very few studies till date have fully dissected this nexus. Nevertheless, the direct clinical approach of administering probiotics can be a novel approach for treating bone loss in osteoporosis via modulating host immunity (Yousf et al., 2015; Britton et al., 2014; Li et al., 2016). This mechanism of probiotics induced blunting of inflammation can thus prove to be an effective and cheap alternative for future therapeutics in the field (Yousf et al., 2015; Britton et al., 2014; Li et al., 2016).

Regulatory T cells (Treg cells) inhibit osteoclastogenesis and are thus known protectors of bone health. Whereas Th17 cells induce osteoclastogenesis and are involved in enhanced bone loss in osteoporosis. Also, Treg cells inhibit the differentiation of Th17 cells and vice-versa (McGovern et al., 2012). This delicate homeostatic balance between Treg-Th17 cells is of utmost importance in various inflammatory conditions. The fact that intestinal microenvironment favours Treg cell generation (Omenetti and Pizarro, 2015) is the focus of our interest in the present study as to how probiotics induced Treg cells would regulate bone health. Surprisingly, very few studies till date have analyzed this connection. Also, the magnitude of Treg cell induction by different probiotics strains has still not been well characterized. A broader interpretation of this question would reveal many possible mechanisms whereby the probiotics may affect bone health. Thus, use of probiotic strains may afford opportunities for new interventions to improve bone health and reduce the risk for osteoporosis and fracture. We selected *Lactobacillus acidophilus* (LA), as probiotic of our choice in the present study, since no study has been done till date to delineate its role in mediating bone health. Also, the effect of LA on Treg-Th17 equilibrium is still not defined. LA is one of the most common probiotic strains available in our homes (curd, yoghurt, kefir). In our present study, we report for the first time that administration of LA due to its immunomodulatory effect on Treg-Th17 cell balance (enhanced Treg and decreased Th17 cells) in vivo, suppresses expression of osteoclastogenic factors (IL-6, IL-17, RANKL and TNF-α) and induces expression of anti-osteoclastogenic factors (IL-10 and IFN-γ) leading to enhanced bone mass. The present study highlights this interesting and novel field of bone biology (termed now as “Osteomicrobiology”) and will have immense clinical implications in near future (Dar et al., 2018; Jones et al., 2017). Administration of various probiotic strains can thus open new avenues in treatment of various inflammatory bone conditions such as osteoporosis and RA.

## 2 Methods

### 2.1 Animals

Thirty female mice (BALB/c) of 8–10 weeks with an average body weight of 25 g were selected. Mice were housed in standard cages, maintained under specific pathogen-free conditions and fed sterilized food and autoclaved water ad libitum. Mice were either ovariectomized (ovx) or sham operated with intra-peritoneal injection of Ketamine (100–150 mg/kg) and Xylazine (5–16 mg/kg) through standard protocol and were grouped under three categories with 10 mice in each group (Group A: No Probiotic + sham operated, Group B: ovx and Group C: ovx + LA). Ovx + LA group received daily oral dose of 200 μl (of 10^9^ cfu/ml *Lactobacillus acidophilus* suspension) constituted in drinking water for a period of 6 weeks post-ovariectomy as per standardized protocols (Ohlsson et al., 2014; Britton et al., 2014), while control mice received normal water. At the end of experiment (6 week), animals were sacrificed for further analysis. All the procedures involving animals were conducted according to the requirements and with the approval of the Institutional Animal Ethics Committee of SIPS.

### 2.2 *Lactobacillus acidophilus* (LA) culture

*Lactobacillus acidophilus* (LA) ATCC 4356 was provided by Dr. Rajesh K. Mondal from Department of Microbiology, Dr. Harisingh Gour Central University, Sagar (MP)-470003, India. Briefly, LA were inoculated in deMan, Rogosa, and Sharpe broth (MRS; Difco) and grown at 37 °C for 20 h, then resuspended in phosphate-buffered saline (PBS) before administering to the mice with 200 μl suspension (10^9^ cfu/ml *Lactobacillus acidophilus*).

### 2.3 Antibodies and reagents

The following antibodies/kits were bought from BD Biosciences (USA): PerCP-Cy-5.5 Rat Anti-Mouse CD4-(RM4-5) (550954), APC-Rat-Anti-Mouse-CD8a-(53-6.7) (561093), PE-Rat-Anti-Mouse-CD45R/B220-(RA3-6B2) (553089) and mouse Cytometric Bead Array-(CBA) kit. The reagents Anti-Human/Mouse Rorγt-PE-(AFKJS-9) (12-6988), Anti-Mouse/Rat Foxp3-APC-(FJK-16s) (17-5773), Foxp3/Transcription factor staining buffer (0-5523-00), RBC lysis buffer (00-4300-54) were obtained from eBioscience (USA). Anti-Mouse-CD254-(TRANCE-RANKL)-PE (IK22/5) (510005), Anti-Mouse-CD8-PE/Cy7 (53-6.7) (100721), Anti-Mouse-IFN-γ-FITC (XMG 1.2) (505805) and Anti-Mouse-IL-17A-PE (TC11-8H10.1) (506903) were obtained from BioLegend (USA). Mouse/Rat Estradiol ELISA Kit (Calbiotech, Spring Valley, CA, USA) and all procedures were followed according to the manufacturer's instruction.

### 2.4 Scanning electron microscopy (SEM)

For SEM analysis, femur cortical bone samples were kept in 1% Triton for 48–72 h and later transferred to 1×PBS solution till final analysis is done. Bone slices were made and dried under incandescent bulb before SEM analysis and scanned in NOVA NANOSEM 450 microscope equipped with a tungsten filament gun operating at WD 10.6 mm and 20 kV. SEM images were digitally photographed at low (15×), intermediate (1000×), and high magnifications (10,000×) to picture the best cortical structure. Images were later processed and analyzed using Adobe Photoshop 7.0. The data was further analyzed using MATLAB software (Mathwork, USA).

### 2.5 Atomic force microscopy (AFM)

Femur cortical bone samples were dried in dust free environment with 60 W lamps for 6 h followed by high vacuum drying and subsequently analyzed by Atomic Force Microscope (INNOVA, ICON, Bruker), operating under the Acoustic AC mode (AAC or Tapping mode). This was assisted with cantilever (NSC 12(c) from MikroMasch, Silicon Nitride Tip) by NanoDrive™ version 8 software at a force constant of 0.6 N/m having resonant frequency at 94–136 kHz. The images were taken in air at room temperature at the scan speed of 1.5–2.2 lines/s. The data analysis was done using of Nanoscope analysis software. The data was further analyzed using MATLAB software (Mathwork, USA).

### 2.6 Micro computed tomography (μ-CT)

μ-CT of the femur/Tibia (trabecular/cortical) and lumbar vertebrae-V (trabecular) was performed using SkyScan 1076 scanner (SkyScan). Scanning was done at 70 kV, 100 mA using a 0.5-mm aluminum filter and exposure set to 590 ms. In total, 1800 projections were collected at a resolution of 6.93 μm/pixel. For purpose of carrying out reconstruction process NRecon software was used. Bone length was determined from the rendered 3D images in CTAn software. The distance from trochanter to edge of femoral condyles defined total femoral length. Tibial length calculated as distance from medial condyle to medial malleolus. Manual segmentation of 2D slice of sagittal images was done to isolate growth plates from the surrounding bone tissue in micro-CT images followed by reconstruction to render 3D images. It was from these images that growth plate height was measured using data viewer software. For trabecular region analysis, ROI was drawn at a total of 100 slices in secondary spongiosa at a distance of 1.5 mm from distal border of growth plates excluding the parts of cortical bone and primary spongiosa. In vivo measurement of LV5 trabecular was done by encompassing 50 continuous slices which start from the beginning of trabecular bone within spinal body (Li et al., 2016; Dempster et al., 2013). The CTAn software was used to analyze cortical bone by taking into consideration 350 consecutive image slides discarded from growth plate to leave out all trabecular regions, out of these 200 continuous images were selected as such. Various bone histomorphometric trabecular parameters (3D) and cortical parameters (2D) were analyzed by already established protocols (Dempster et al., 2013; Srivastava et al., 2013). For determination of BMD of LV5, femur and tibia μ-CT scans were utilized as determined from VOI made for trabecular and cortical region. For BMD calibration, 2-mm-diameter hydroxyapatite phantom rods with known BMD (0.25 and 0.75 g/cm^3^) were used. For each analysis, the estimated BMD was determined based on linear correlation between the μCT attenuation coefficient and BMD (Srivastava et al., 2013).

### 2.7 FTIR

The FTIR analysis of femur cortical bones was done by using 8400S-Fourier Transform Infrared Spectrophotometer (SHIMADZU), working on resolution 4 cm^−1^; scan speed 2.5 kHz, and 128 scans co-addition, in the KBr pellet form. With the aid of Savitzky-Golay algorithm noise was eliminated to obtain smooth spectra of samples. The samples were held in contact with a prism made of highly refractive material transmitting infrared rays which are made incident on the sample at angle that induces total reflection. Bones were kept in 1% Triton, washed after 24 h and dried under 60 W lamps for 6 h followed by high vacuum drying. Dry bones were made into powder form and mixed with KBr (potassium bromide) in 1:100 ratios and analyzed for FTIR.

### 2.8 Flow cytometry

T cells in single-cell suspensions isolated from bone marrow and spleen were stained with PerCP-Cy5.5-anti-CD4 and PE/Cy-7-anti-CD8 or APC-anti-CD8. Cells were allowed to pellet down after centrifugation at 1800 rpm for 5 min followed by addition of 1 ml RBC lysis buffer. Cells were washed thoroughly with wash buffer and fixed-permeabilized with Foxp3 permeabilization buffer for 30 min on ice. Intracellular Foxp3, RANKL and Rorγt staining was performed using APC-conjugated anti-Foxp3, PE-conjugated anti-RANKL and PE-conjugated anti-Rorγt Abs in permeabilization buffer for 45 min on ice. Cells were washed thoroughly with permeabilization buffer and analyzed by flow cytometry.

Intracellular staining of Tregs and Th17 cells was performed by stimulating cells for 5-6 h with PMA (50 ng/ml) and ionomycin (500 ng/ml). After 1 h, GolgiStop (1 μg/ml) was added in the cultures to block cytokine secretion. Cells were first surface stained with PerCP-Cy5.5-anti-CD4 and PE/Cy-7-anti-CD8 antibodies for 30 min on ice. Cells were washed thoroughly with wash buffer and further fixed-permeabilized with Foxp3 fixation-permeabilization buffer for 30 min on ice. Cells were washed thoroughly with Foxp3-permeabilization buffer and stained intracellularly with APC conjugated anti-Foxp3 and PE-conjugated anti-IFN-γ; FITC-conjugated anti-IL-10 and PE-conjugated IL-17A antibodies in permeabilization buffer for 30 min on ice (Srivastava et al., 2011). Cells were washed thoroughly with Foxp3-permeabilization buffer and analyzed by flow cytometry on BD FACS ARIA III (BD Biosciences), and the data was analyzed using FloJo (TreeStar, USA) software. Results are expressed as cell frequencies.

### 2.9 Cytometric bead array (CBA)

Levels of IL-6, IL-10, IL-17A, IFN-γ and TNF-α in blood serum were assessed by fluorescent bead-based technology using CBA kit and all procedures were followed according to the manufacturer's instruction (BD Biosciences, USA). Fluorescent signals were read and analyzed on a FACS Aria III flow cytometer (BD Biosciences) with the help of BD FCAP Array software (BD Biosciences).

### 2.10 Statistical analysis

The results were evaluated by using ANOVA with subsequent comparisons by Student *t*-test for paired or nonpaired data as appropriate. Analysis was performed using Sigma plot software (Systat Software, Inc., Germany). Values are reported as mean ± SEM (*n* = 10) and similar results were obtained in three independent experiments. Statistical significance was defined as *p* ≤ 0.05 (*p ≤ 0.05, **p ≤ 0.001, ***p ≤ 0.0001) with respect to ovx mice group.

## 3 Results

### 3.1 LA administration inhibits bone loss

**Fig. 1:**
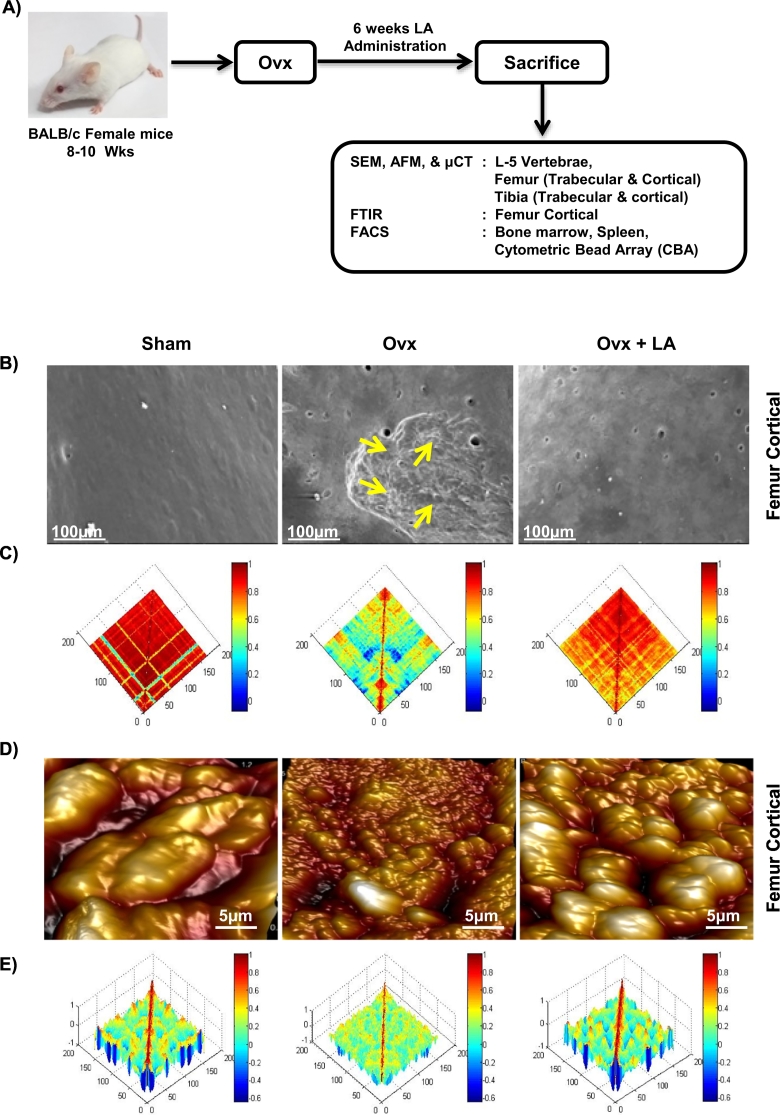
LA inhibits bone loss. A) Experimental work plan. Ovx + LA group received 200 μl of 10^9^ cfu/ml *Lactobacillus acidophilus* orally constituted in drinking water. At the end of 6^th^ week post-ovariectomy mice were sacrificed and cortical bones of sham, ovx and ovx + LA groups were collected for SEM and AFM analysis. B) 2D SEM images. C) 2D MATLAB analysis of SEM images. D) 3D AFM images. E) 3D MATLAB analysis of AFM images. The above images are representative of one experiment and similar results were obtained in three different experiments with *n* = 10 mice/group/experiment.

Ovx status was routinely verified for every batch of ovariectomized mice by Estradiol (E2) estimation from the serum samples of sham and ovx groups using enzyme-linked immunosorbent assay as per manufacturer's instruction. Notably, we consistently observed significantly decreased levels of estradiol (E2) in ovx mice as compared to sham groups (Supplementary Fig. S1). To determine the effect of LA administration on bone health we were first interested in looking for different signs of bone remodelling by scanning the surface of bones by SEM/AFM. To determine the same, mice were divided in three group's viz. sham, ovx and ovx + LA and at the end of the experiment (6^th^ week post-ovariectomy), mice were sacrificed, and femur bone collected for SEM (Fig. 1A). SEM images clearly indicated a significantly higher number of resorption pits/lacunae (increased osteoclastogenesis) of various shapes/area on the surface of the bones in ovx group with respect to sham group. Strikingly these resorption pits/lacunae were either significantly absent or were in very few numbers in ovx + LA administered groups (Fig. 1B). AFM is a powerful tool for imaging of bone ultra-structure in a close to physiological state (Thurner et al., 2007), with detailed understanding of the influence of the remodelling on the structure of bone and provides a better understanding of the bone surface topology. As expected our AFM images complemented our results from SEM by revealing significantly decreased number of scalloped surfaces or pits (Fig. 1D) left behind by the osteoclasts in ovx + LA administered group in comparison to both sham and ovx group.

(Mouse image courtesy: Hamid Y. Dar).

MATLAB (matrix laboratory) is a multi-paradigm numerical computing technique used in microscopy, biomedical imaging etc. We thus performed MATLAB analysis of both SEM and AFM images to further support our data, which shows the correlation between bone loss and bone mass. Any distortion in the object or absence of identical object reduces the correlation value and is usually represented through colours. It can be clearly concluded from Fig. 1C (2D-SEM) that sham and ovx + LA groups have similar structures while that of ovx is very much degraded (enhanced osteoclastogenesis). The MATLAB-analysis (Fig. 1C) shows this extent of homogeneity observed in the SEM images by red (highest correlation-more bone) and blue (least correlation-less bone). Fig. 1D (3D-AFM) further complements our results that both sham and ovx + LA images are very much similar (normal bone topology with high bone content) whereas the ovx image is having different topology (eaten-up bone surface due to enhanced osteoclastogenesis). In MATLAB-analysis (Fig. 1E), red implies height of bone (minimum osteoclastogenesis) and the representative valleys-blue describe decreased bone height (enhanced osteoclastogenesis). The representative images in Fig. 1 corroborate results of one independent experiment and similar images/results were obtained in three independent experiments with 10 mice/group/experiment. Taken together, both SEM and AFM analysis of bone samples clearly indicate that administration of LA inhibits osteoclastogenesis mediated bone loss thereby resulting in enhanced bone mass.

### 3.2 LA administration enhances lumbar vertebral bone micro-architecture in ovx mice

**Fig. 2:**
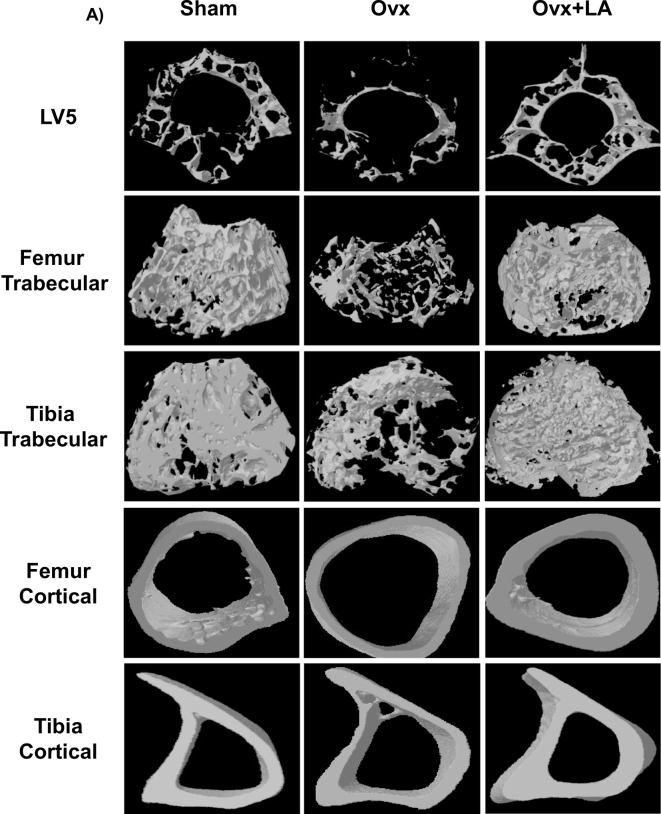
LA administration enhances trabecular and cortical bone microarchitecture. A) 3-D μCT reconstructions of LV5, femur trabecular, tibia trabecular, femur cortical and tibia cortical of sham, ovx and ovx + LA groups. The above images are representative of one experiment and similar results were obtained in three different experiments with *n* = 10 mice/group/experiment.

Moving ahead in our study we were next interested in delineating the effect of LA intake on bone histomorphometry. We thus performed μCT (a “gold standard” for evaluation of bone morphology and microarchitecture) for quantitating various bone morphometric and geometric parameters related to bone loss. Since the lumbar vertebrae-5 (LV-5) is considered as one of the best diagnostic parameters to study the effect of bone loss via μCT in osteoporosis, we too were interested in analysing the effect of LA intake on LV-5 vertebrae. Strikingly, μCT analysis clearly pointed towards a significant improvement of the LV-5 microarchitecture in LA administered group with respect to both sham and ovx group (Fig. 2). We observed that in comparison to ovx group, the ovx + LA administered group had increased bone volume/tissue volume (BV/TV) (*p* < 0.01), trabecular thickness (Tb. Th) (*p* < 0.01), trabecular number (Tb. N) (*p* < 0.0001) and connectivity density (Conn. Den.) (*p* < 0.001) (Table 1); whereas on the other hand in comparison to ovx group the ovx + LA administered group had decreased trabecular separation (Tb. Sp.) (*p* < 0.01) and trabecular pattern factor (Tb. Pf.) (*p* < 0.01) (Table 1). Together our data clearly suggest that LA administration significantly inhibits bone loss by enhancing various trabecular parameters in LV-5 vertebrae of post-menopausal osteoporotic mice model.

**Table 1:** Bone histomorphometric parameters of trabecular and cortical bones. LA enhances both trabecular and cortical bone microarchitecture. Histomorphometric parameters of trabecular and cortical bones of LV5, femur and tibia. Bone volume/tissue volume ratio (BV/TV); Tb. Th., trabecular thickness; Tb. No., trabecular number; Conn. Den., Connectivity density; Tb. Sp., trabecular separation; Tb. Pf., trabecular pattern factor; Tt. Ar., total cross-sectional area; T. Pm., total cross-sectional perimeter; Ct. Ar., cortical bone area; B. Pm., bone perimeter; Ct. Th., average cortical thickness; MMI., polar moment of inertia. The results were evaluated by using ANOVA with subsequent comparisons by Student *t*-test for paired or nonpaired data as appropriate. Analysis was performed using Sigma plot software (Systat Software, Inc., Germany). Inner values are reported as mean ± SEM (*n* = 10) and similar results were obtained in three independent experiments. Statistical significance was defined as *p* ≤ 0.05 (*p ≤ 0.05, **p ≤ 0.001, ***p ≤ 0.0001) with respect to ovx mice group.

Bone parameters	Sham	Ovx	Ovx + LA
LV5			
BV/TV (%)	23.5 ± 0.04	16.53 ± 1.61	21.83 ± 0.04**
Tb. Th (mm)	1.65 ± 0.08	0.64 ± 0.16	1.42 ± 0.07**
Tb. No (mm^−1^)	4.67 ± 0.52	1.78 ± 0.04	4.15 ± 0.20***
Conn. Den (mm^−3^)	6.83 ± 1.34	2.78 ± 0.97	6.11 ± 1.22**
Tb. Sp. (mm)	1.32 ± 0.08	2.54 ± 0.24	1.65 ± 0.04**
Cl. Po (%)	8.15 ± 0.07	10.87 ± 0.24	8.21 ± 0.16**

Femur trabecular			
BV/TV (%)	30.71 ± 0.32	13.21 ± 3.21	25.41 ± 1.51**
Tb. Th (mm)	1.73 ± 0.14	0.74 ± 0.16	1.45 ± 0.08**
Tb. No (mm^−1^)	5.21 ± 0.11	2.33 ± 0.43	4.76 ± 0.08**
Conn. Den (mm^−3^)	9.64 ± 0.68	4.51 ± 1.04	7.95 ± 0.06**
Tb. Sp. (mm)	1.60 ± 0.51	3.84 ± 0.06	1.86 ± 0.08**
Cl. Po (%)	3.02 ± 0.09	5.45 ± 0.21	3.32 ± 0.13**

Tibia trabecular			
BV/TV (%)	26.67 ± 0.35	20.11 ± 0.35	25.31 ± 0.24**
Tb. Th (mm)	1.88 ± 0.02	1.10 ± 0.02	1.76 ± 0.03*
Tb. No (mm^−1^)	4.60 ± 0.17	3.01 ± 0.48	4.25 ± 0.15**
Conn. Den (mm^−3^)	4.45 ± 0.77	2.58 ± 0.76	4.26 ± 0.08**
Tb. Sp. (mm)	1.87 ± 0.23	3.37 ± 0.07	2.10 ± 0.12**
Cl. Po (%)	2.76 ± 0.04	4.96 ± 0.61	3.12 ± 0.25**

Femur cortical			
Tt. Ar (mm^2^)	2.76 ± 0.02	1.18 ± 0.10	2.55 ± 0.11**
T. Pm (mm)	7.65 ± 1.21	4.22 ± 0.17	6.43 ± 0.07**
Ct. Ar (mm^2^)	8.98 ± 0.21	3.51 ± 0.48	7.05 ± 0.11**
B. Pm (mm)	1.12 ± 0.07	0.64 ± 0.10	1.10 ± 0.09**
Ct. Th (mm)	1.22 ± 0.01	0.54 ± 0.06	1.09 ± 0.59**
MMI (mm^4^)	2.42 ± 0.12	1.21 ± 0.25	2.24 ± 0.17**

Tibia cortical			
Tt. Ar (mm^2^)	4.88 ± 0.06	3.12 ± 0.91	4.80 ± 0.0.98**
T. Pm (mm)	7.19 ± 0.12	5.12 ± 0.97	6.98 ± 0.57*
Ct. Ar (mm^2^)	4.93 ± 0.32	3.18 ± 0.57	4.76 ± 0.32*
B. Pm (mm)	8.69 ± 0.77	6.31 ± 0.14	8.23 ± 0.24*
Ct. Th (mm)	7.78 ± 0.11	5.03 ± 0.89	7.43 ± 0.29***
MMI (mm^4^)	4.53 ± 0.67	3.12 ± 0.35	4.23 ± 0.52*

### 3.3 LA enhances femoral and tibial architecture in ovx mice

Since bone micro-architecture consists of both the trabecular and cortical parameters, we were interested in quantitating both separately in case of femur and tibia. In gross observation by 3D-μCT of femur/tibia bones collected for μCT analysis, significant improvement in the femur trabecular architecture was readily observed in LA treated group when compared with ovx group (Fig. 2). The ovx + LA has increased BV/TV (*p* < 0.01), Tb. Th (p < 0.01), Tb. N (*p* < 0.01) and Conn. Den (*p* < 0.01) (Table 1); but decreased Tb. Sp (*p* < 0.01) and Tb. Pf (*p* < 0.01) (Table 1) in comparison to ovx group. Similar results were also observed in femur cortical bones (Table 1) with significant increase in the cortical micro-architecture in ovx + LA administered group as compared with ovx group. Further our data of μCT analysis of tibia trabecular micro-architecture of LA group showed a clear and significant improvement even in trabecular bone of tibia as compared with ovx group (Fig. 2). The ovx + LA administered group has elevated BV/TV (*p* < 0.01), Tb. Th (*p* < 0.05), Tb. N (*p* < 0.01) and Conn. Dn (*p* < 0.01) along with decreased Tb. Sp (*p* < 0.01) and Tb. Pf (*p* < 0.01) (Table 1) in comparison to ovx group. Similar results were also observed in tibia cortical bones (Table 1), with significant enhancement of cortical bone micro-architecture in ovx + LA administered group as compared to ovx group. Altogether, these results further support and strengthen our observations that administration of LA in ovx mice enhances both femoral and tibial bone micro-architecture.

### 3.4 LA improves both mineral density and heterogeneity of bones

Bone mineral density (BMD) defines the quantity of mineralized tissue present (size and density) in bones and is usually considered for determining tendency of bones to undergo fracture. Thus, we were excited to next determine the effect of LA administration on BMD for both cortical and trabecular bones. Interestingly, both trabecular and cortical bones from femur/tibia/LV5 illustrated a significant increase in their respective BMDs. The BMDs of ovx + LA administered group was significantly enhanced as compared to ovx group in LV-5-trabecular (*p* < 0.01), femur trabecular (*p* < 0.01), tibia trabecular (*p* < 0.05), tibia cortical (*p* < 0.05) and femur cortical (*p* < 0.05) (Table 2A). The composition of bone is heterogeneous due to its constant remodelling and mineralization cycles. Since loss of heterogeneity in bones has been associated with severe fractures and osteoporosis (Boskey, 2015), it thus makes sense to study the effect of LA administration on bone heterogeneity parameters viz. crystallinity (XST), mineral to organic matter ratio (m/m) and carbonate content (c/p) (Boskey, 2015; Gadeleta et al., 2000). Thus, we next investigated the effect of LA administration on heterogeneity of bone content with the help of FTIR analysis. Mice were sacrificed at the end of experiment and femur cortical bones were collected for analysis of heterogeneity. Interestingly, we observed that LA administration significantly enhanced heterogeneity of the bones. Thus, administration of LA in ovx mice was found to significantly reduce the m/m ratio (*p* < 0.01), XST (*p* < 0.005) and c/p ratio (*p* < 0.05) in femur cortical bones with respect to ovx group (Table 2B). In summary, these data clearly point to the novel role of LA in maintaining the natural physiology of bones even in ovx mice, thus making them a suitable therapeutic option with respect to currently available drugs (e.g. bisphosphonates etc.).

**Table 2:** BMD and heterogeneity of bones.

A) BMD of trabecular and cortical bones					
	LV5 (gm HA/cm^3^)	Femur trabecular (gm HA/cm^3^)	Tibia trabecular (gm HA/cm^3^)	Femur cortical (gm HA/cm^3^)	Tibia cortical (gm HA/cm^3^)
Sham	2.14 ± 0.07	3.23 ± 0.08	3.62 ± 0.58	0.91 ± 0.02	0.99 ± 0.012
Ovx	1.74 ± 0.20	1.77 ± 0.40	2.77 ± 0.19	1.02 ± 0.016	1 ± 0.03
Ovx + LA	2.8 ± 0.08**	3.54 ± 0.24**	3.51 ± 0.24*	1.12 ± 0.02*	1.11 ± 0.03*


B) Compositional changes in cortical bones			
	Mineral/matrix ratio (m/m)	Crystallinity (XST)	Carbonate/phosphate ratio (c/p)
Sham	7.19 ± 0.01	4.37 ± 0.07	6.91 ± 0.11
Ovx	7.8 ± 0.05	5.94 ± 0.06	7.56 ± 0.03
Ovx + LA	6.7 ± 0.1**	4.57 ± 0.12*	7.03 ± 0.02*

### 3.5 LA enhances bone mass by modulating Treg-Th17 cell balance

**Fig. 3:**
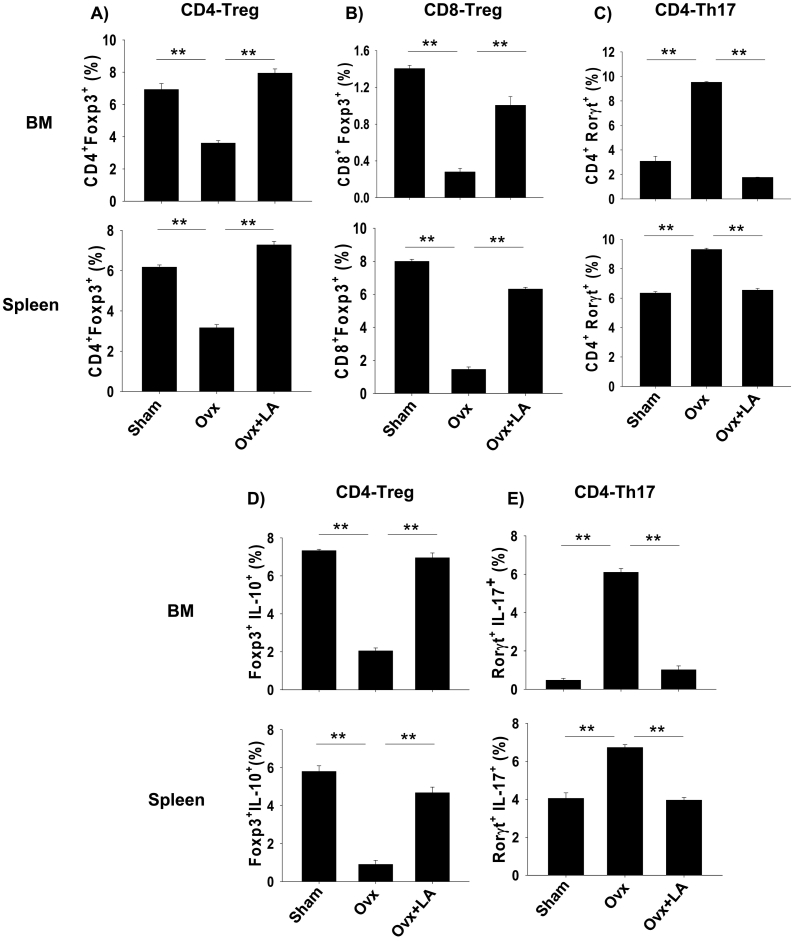
LA modulates Treg-Th17 cell balance in vivo. Cells from bone marrow (BM) and spleen of sham, ovx and ovx + LA mice groups were isolated at the end of experiment, labelled and analyzed by Flow cytometry for percentage of CD4^+^Foxp3^+^, CD4^+^Rorγt^+^, CD4^+^IL-10^+^ Foxp3^+^ and CD4^+^Rorγt^+^IL-17^+^ cells. Gating was first performed for CD4^+^ and later analyzed for the expression of CD4^+^Foxp3^+^, CD4^+^Rorγt^+^, CD4^+^ IL-10^+^ (Tregs) and CD4^+^ IL-17^+^ (Th17) cells. A) Average percentage (%) of CD4^+^Foxp3^+^T cells. B) Average percentage (%) of CD8^+^Foxp3^+^T cells. C) Average percentage (%) of CD4^+^Rorγt^+^T cells. D) Average percentage (%) of Foxp3^+^ IL-10^+^ (Tregs) cells. E) Average percentage (%) of Rorγt^+^IL-17^+^ (Th17) cells. The results were evaluated by using ANOVA with subsequent comparisons by Student *t*-test for paired or nonpaired data, as appropriate. Analysis was performed using Sigma plot software (Systat Software, Inc., Germany). Values are reported as mean ± SEM (*n* = 10) and similar results were obtained in three independent experiments. Statistical significance was defined as *p* ≤ 0.05 (*p ≤ 0.05, **p ≤ 0.001) with respect to ovx mice group.

The role of Treg and Th17 cells in modulating bone health is now well established. Thus, to delineate the role of Treg and Th17 cells in LA modulated bone health we carried out flow cytometric analysis of bone marrow and spleen for both Foxp3^+^ and Rorγt^+^ master-transcription factors for Treg and Th17 cells respectively. Mice were sacrificed after completion of 6 weeks of LA administration, and total lymphocyte populations derived from bone marrow and spleen were analyzed for percentage of CD4^+^Foxp3^+^Treg cells, CD8^+^Foxp3^+^Treg cells and CD4^+^Rorγt^+^Th17 cells by flow cytometry. Gating strategy for CD4^+^Foxp3^+^Treg cells, CD8^+^Foxp3^+^Treg cells and CD4^+^Rorγt^+^Th17 in bone marrow and spleen was performed as per representative figures in Supplementary Figs. S2 and S3 respectively. As compared with ovx mice, LA administration in ovx mice led to 1.5-fold increase in CD4^+^Foxp3^+^Treg cells in bone marrow (3.42 ± 2% in ovx to 8.20 ± 1% in LA) (*p* < 0.01) and one-fold increase in spleen (3.17 ± 2% in ovx to 7.38 ± 1.5% in LA) (*p* < 0.01) (Fig. 3A). Interestingly, LA administration also led to 4-fold increase in CD8^+^Foxp3^+^ Treg cells in bone marrow (0.3 ± 0.01% in ovx to 1.2 ± 0.02% in LA) (*p* < 0.01) and three-fold increase in spleen (1.83 ± 0.42% in ovx to 7.08 ± 0.86% in LA) (*p* < 0.01) (Fig. 3B) as compared to ovx mice. Next, we determined the effect of LA administration on Th17 cells, strikingly in LA administered group, we observed ≥ three-fold decrease in percentage of CD4^+^Rorγt^+^Th17 cells in bone marrow (9.4 ± 0.63% in ovx to 2.11 ± 0.03% in LA) (*p* < 0.01) and >1.5-fold decrease in spleen (9.76 ± 0.56% in ovx to 6.21 ± 0.41% in LA) (*p* < 0.01) (Fig. 3C) with respect to ovx mice.

It is now well established that IL-10 cytokine possess anti-inflammatory properties and is responsible for regulatory functions of Tregs during inflammation (Li et al., 2016). On the other hand, Th17 cells produce IL-17, responsible for enhanced osteoclastogenesis in ovx induced osteoporosis (Kotake et al., 1999; Adamopoulos et al., 2010). Our next aim was to investigate the expression of IL-10 and IL-17 by Treg and Th17 cells respectively in both bone marrow and spleen. Therefore, to explore the source of IL-10 and IL-17 secretion, total lymphocytes from spleen and bone marrow were activated with PMA (50 ng/ml) and ionomycin (500 ng/ml) for 5–6 h before analysis for the secretion of IL-10 and IL-17 by Flow cytometry. Gating was performed first for CD4^+^ and later analyzed for the expression of both Foxp3^+^IL-10^+^ (Treg) cells and Rorγt^+^IL-17^+^ (Th17) cells. We observed that LA administration in ovx mice led to >1.5-fold increase in Foxp3^+^IL-10^+^ cells in bone marrow (2.01 ± 0.42% in ovx to 7.34 ± 0.78% in LA) (*p* < 0.001) and an increase of close to two-fold in spleen (1.78 ± 0.45% in ovx to 4.32 ± 0.43% in LA) (*p* < 0.01) (Fig. 3D) along with four-fold decrease in Rorγt^+^IL-17^+^ cells in bone marrow (6.11 ± 0.78% in ovx to 1.35 ± 0.12% in LA) (*p* < 0.01) and >1.5-fold in spleen (7.23 ± 0.45% in ovx to 3.87 ± 0.32% in LA) (*p* < 0.01) (Fig. 3E). Collectively, these results suggest that LA enhances production of IL-10 from Tregs along with simultaneously inhibiting the expression of IL-17 from Th17 cells. Thus, our results clearly suggest that LA has potential to augment Treg-Th17 cell axis by increasing the percentage of Foxp3^+^ Treg cells (both CD4 and CD8) and inhibiting Th17 cells in vivo.

### 3.6 LA administration skews expression of osteoclastogenic factors

**Fig. 4:**
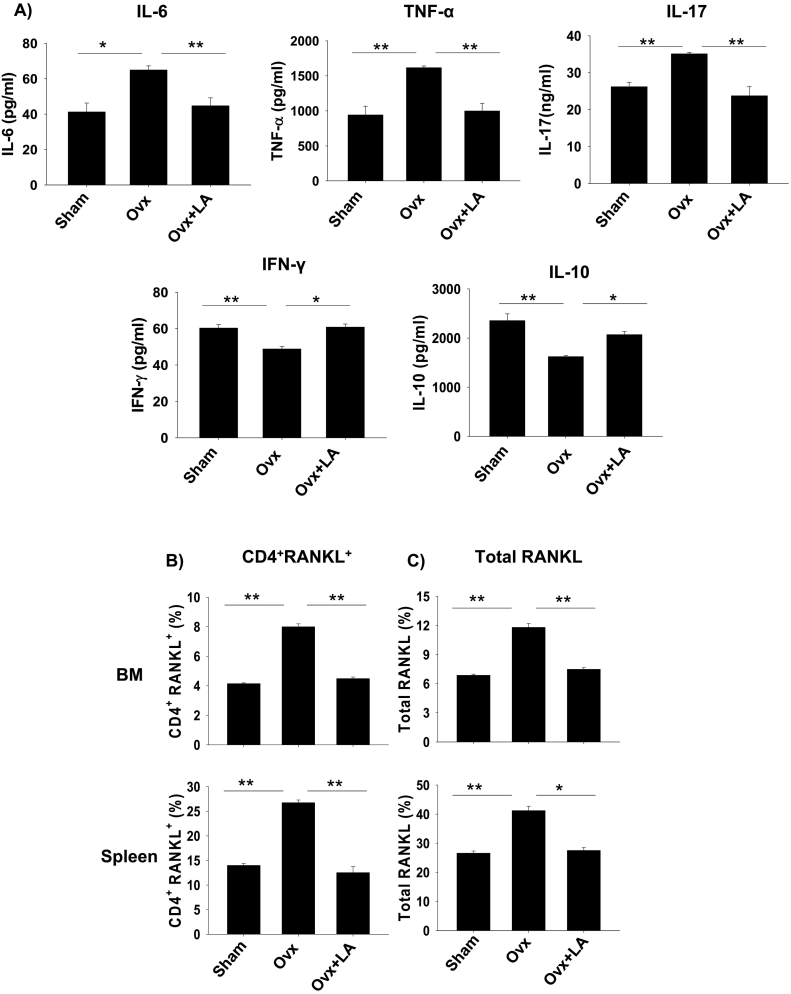
LA skews balance of osteoclastogenic factors in ovx mice. A) Serum samples of mice were analyzed for secretion of various proinflammatory and anti-inflammatory cytokines by CBA. B) Average percentage (%) of CD4^+^RANKL^+^T cells. C) Average percentage (%) of total RANKL. The results were evaluated by using ANOVA with subsequent comparisons by Student *t*-test for paired or nonpaired data, as appropriate. Analysis was performed using Sigma plot software (Systat Software, Inc., Germany). Values are reported as mean ± SEM (*n* = 10) and similar results were obtained in three independent experiments. Statistical significance was defined as *p* ≤ 0.05 (*p ≤ 0.05, **p ≤ 0.001) with respect to ovx mice group.

**Fig. 5:**
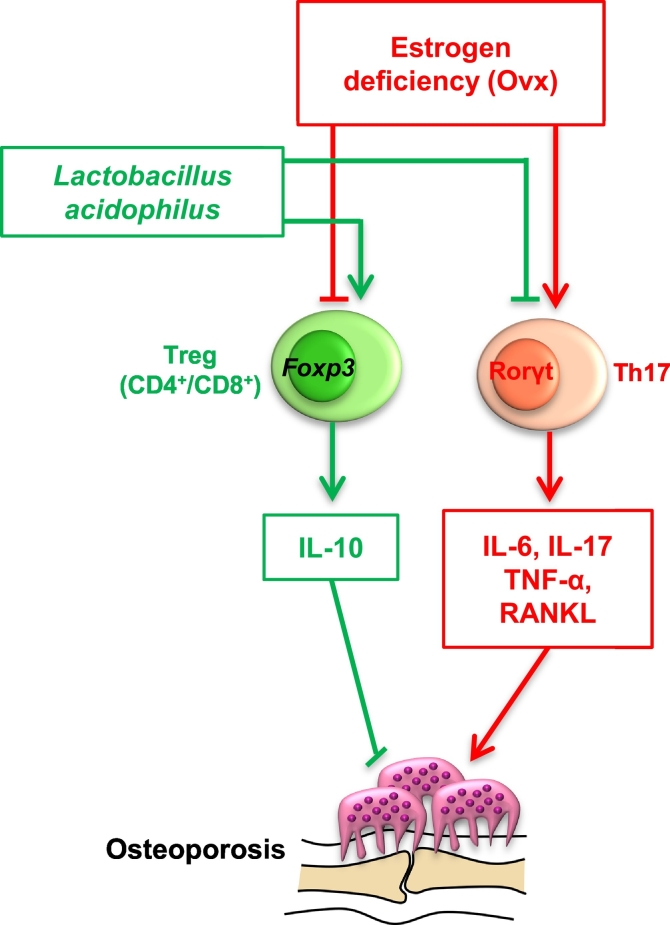
Summary of our results. LA administration enhances bone mass by skewing Treg-Th17 balance in post-menopausal osteoporotic (ovx) mice.

The role of various pro-inflammatory cytokines in the pathogenesis of various inflammatory diseases is well established. Also, various studies have mentioned the negative effect of pro-inflammatory cytokines on bone health leading to various diseases such as osteoporosis, osteoarthritis etc. (D'Amelio et al., 2008). To determine the same, mice were sacrificed at the end of the experiment and blood serum cytokines level was analyzed by either CBA. It was observed that LA administration results in significant decrease in levels of osteoclastogenic factors viz. IL-6 (*p* < 0.01), TNF-α (*p* < 0.01) and IL-17A (*p* < 0.01) in comparison to ovx group (Fig. 4A). Interestingly, LA administration also significantly increased the levels of IFN-γ (*p* < 0.05) and IL-10 (*p* < 0.01) which are known anti-osteoclastogenic factors/cytokines (Zhang et al., 2011). RANKL is one of the prime factors for inducing osteoclastogenesis. RANKL has been reported as the prime factor for inducing osteoclastogenesis and is known to be expressed by activated T cells (including Th17 cells) (Zhang et al., 2011). Thus, it's important to determine the amount and source of RANKL in our system. In line with this we next examined the amount of total RANKL produced by all sources, along with RANKL secreted by T cells alone. Strikingly, administration of LA in ovx mice caused a two-fold reduction in RANKL secreting CD4^+^T cells in bone marrow (8.12 ± 0.75% in ovx to 4.01 ± 0.32% in LA) (*p* < 0.01) along with two-fold reduction in spleen (26.32 ± 1.22% in ovx to 13.75 ± 2.11% in LA) (*p* < 0.01) with respect to ovx mice (Fig. 4B). Notably, it was observed that the total RANKL expression was also decreased significantly in ovx + LA administered group in bone marrow (11.88 ± 0.67% in ovx to 7.23 ± 0.44% in LA) (*p* < 0.01) and more than one-fold decrease in spleen (42.3 ± 2% in ovx to 26.1 ± 1% in LA) (*p* < 0.05) with respect to ovx mice (Fig. 4C). Collectively our results confirm that LA administration inhibits production of proinflammatory factors/cytokines and facilitate secretion of anti-inflammatory cytokines, thereby corroborating inflammatory bone loss in ovx-mice (Fig. 5).

## 4 Discussion

Menopause is a major risk factor for osteoporosis thereby leading to increased risk of bone fractures and disability. The disease stems mainly from the cessation of ovarian function, where declining estrogen levels result in the stimulation of bone resorption and to a lesser extent bone formation leading to a period of rapid bone loss (Li et al., 2016). Osteoporosis is one of the most important but often neglected bone disease associated with aging and postmenopausal condition. Different drugs have been designed for the treatment of post-menopausal osteoporosis with varying results. Considerable safety concerns are associated with treatment, as currently available drugs (bisphosphonates, strontium, and estrogen-replacement-therapy) for the treatment of osteoporosis are associated with several adverse effects (Yang et al., 2014; Chan et al., 2003; Committee, 2013). With the advent of probiotics boom in the research fraternity in the last few years, probiotics are now being employed as novel therapeutics for treatment of various inflammatory diseases such as IBD, colitis, RA, including osteoporosis (Yousf et al., 2015; Ohlsson et al., 2014; Britton et al., 2014; Li et al., 2016). Probiotics are thus proving to be very safe and efficient way of treating different bone and other health related issues without any substantial side effects (Ohlsson et al., 2014; Britton et al., 2014; Li et al., 2016). This approach can be highly invaluable for prevention of increased bone destruction in patients where inflammation and bone loss cannot be prevented by established older methods.

Various probiotic strains of bacteria have been employed for harnessing their benefits on the host immune system. It has recently been reported that administration of *Lactobacillus paracasei* (NTU 101) and *Lactobacillus plantarum* (NTU 102) fermented milk to ovx mice resulted in higher trabecular number (Chiang and Pan, 2011). *Lactobacillus helveticus* enhances BMD and BMC in osteoporotic rats (Narva et al., 2004). Similarly, *Lactobacillus casei*, *Lactobacillus reuteri*, *Lactobacillus gasseri* and *Bifidobacterium longum* enhanced bone weight (Scholz-Ahrens et al., 2007). A recent study showed a significant reduction in bone resorption by *Lactobacillus reuteri* via decreasing levels of TNF (Britton et al., 2014; Li et al., 2016). These studies have suggested that probiotics can increase bone mass and help reduce osteoporosis by different ways, but the exact mechanism of the same is still unclear. Also, no study has been done on LA strain for regulating bone health and our study elucidates this novel role of LA via modulation of Treg-Th17 cell balance. Importantly, no study till date had unravelled the role of Treg-Th17 cells in probiotics mediated bone health. Thus, to delineate this nexus between LA and bone via its effect on the host immunity, we selected LA strain for studying its effect on bone health in ovx induced osteoporotic mice model. We specifically chose LA as a probiotic strain of our interest due to its easy availability along with its rich association with our diet in various cultures (curd, yoghurt, kefir, etc.) around the world from time immemorial. Our present study demonstrates that oral administration of the probiotic LA enhances bone health even under estrogen deficient conditions. Thus, to elucidate the mechanism of LA in regulating bone health we started with its effect on host physiology, particularly the osteoimmune system. SEM and AFM analysis of bone samples confirmed that administration of LA inhibits bone loss in postmenopausal osteoporotic model in mice. μCT of bones further reaffirmed our results, as significant bone loss was observed in LV-5, femur and tibial bones of ovx mice which was further supported by MATLAB analysis. These results clearly validate and warrant the use of LA as a potent anti-osteoporotic agent.

Bone is a dynamic and heterogeneous tissue consisting of different constituents viz. mineral phase (hydroxyapatite), organic phase (~90% collagen type I, ~5% noncollagenous proteins, ~2% lipids) and water (Boskey, 2015; Gadeleta et al., 2000). With the advancement of age, gender and disease treatment, their relative proportions in the bone vary significantly. Most of the anti-resorptive drug candidates (bisphosphonates, calcitonin, cathepsin K inhibitor, estrogen) available today decrease the heterogeneity of the bone samples. Loss of material heterogeneity is associated with an increase in brittleness thereby increasing the risk of fracture (Boskey, 2015). Thus, the physiological composition of the bone in the healthy individual must be maintained for the proper functioning of the bone. Importantly our results for the first time clearly indicate increase in both the BMD and bone heterogeneity (decrease in m/m, c/p ratio and XST) of bone samples in LA administered ovx mice with respect to ovx mice. These data clearly highlight the novel role of LA in inhibiting bone loss in osteoporosis without even compromising on part of bone heterogeneity. Thus, our data for the first time demonstrate the importance of probiotics in bone health which is at par and even better than the currently available drug candidates (e.g. bisphosphonates-which decreases heterogeneity leading to increased fracture risks in long run) with respect to bone heterogeneity (Boskey, 2015).

Since, LA has been associated with various immunomodulatory properties (De Roock et al., 2009); we next explored the role of LA in modulation of Treg-Th17 cell equilibrium. CD4^+^T cells have established role in inflammatory bone loss (Fujita et al., 1984). Also, the family of T-cells has grown to include Treg and Th17 cells. Treg cells (both CD4^+^ and CD8^+^) are associated with bone protective functions (Luo et al., 2011; Sato et al., 2006; Anderson et al., 2004), whereas Th17 has now been associated with inflammatory bone loss in RA, osteoporosis etc. (Luo et al., 2011). Several strains of *Lactobacillus* have been reported to have therapeutic effect in experimental mouse models of inflammatory bowel disease, atopic dermatitis, and rheumatoid arthritis, and are associated with enrichment of Treg cells in the inflamed regions (Kwon et al., 2010). LA has already been reported to suppress Th17 cells in allergic mice (Kwon et al., 2010; Wen et al., 2012), along with increasing Tregs in colitis mice model (Petersen et al., 2012). Among the mechanisms responsible for bone loss induced by surgical menopause in mice and humans is an expansion of Th17 cells (Khosla and Pacifici, 2013) along with inhibition of Treg cells (Khosla and Pacifici, 2013; Basu et al., 2013). In the present study, we report for the first time that administration of LA in ovx mice significantly increases the population of Tregs (both CD4^+^ and CD8^+^ Treg) and inhibits Th17 cells in bone marrow, a primary site of osteoclastogenesis. Taken together these results clearly demonstrate the immunomodulatory role of LA in enhancing bone health by inhibiting osteoclastogenic Th17 cells and promoting anti-osteoclastogenic Treg cells.

Postmenopausal bone loss is regarded as an inflammatory condition because of the causal role of the immune system (Khosla and Pacifici, 2013; Weitzmann and Ofotokun, 2016) and the presence of increased levels of inflammatory cytokines (Weitzmann and Ofotokun, 2016). Estrogen deficiency inhibits secretion of anti-osteoclastogenic factors/cytokines viz. IL-10 (Eghbali-fatourechi et al., 2003) and IFN-γ (Takayanagi et al., 2000), along with simultaneous induction of IL-6, IL-17, TNF-α and RANKL in ovx mice (Li et al., 2016; Sato et al., 2006; Khosla and Pacifici, 2013; Takayanagi et al., 2000). In our present study, we too report that LA administration in sex steroid deficiency induced osteoporotic mice model, inhibits secretion of inflammatory cytokines such as IL-6, IL-17 and TNF-α and increases the secretion of anti-osteoclastogenic cytokine such as IFN-γ and IL-10. In addition, significant decrease in the production of RANKL from both T cells and non-T cell sources was observed in ovx + LA administered group with respect to ovx mice. Taken together, the present findings clearly demonstrate the anti-inflammatory properties of LA intake in ovx mice by inhibiting osteoclastogenesis and thereby enhancing bone mass.

The positive effects of LA intake on bone health in the present study are very exciting and promising, since LA can be even started as preventive therapeutics in case of both normal and aged individuals, without waiting for any clinical trials which account for huge economic burden and time lag. One of the most prominent features of our study is the observation that supplementation of LA (a known probiotic) entirely protected ovx mice against bone loss and results in a net gain of bone mass in sex steroid deficient mice model. Consistent with these physiological findings, LA administration skews Treg-Th17 cell balance and dampens the levels of osteoclastogenic cytokines following sex steroid depletion. The development of osteoporosis can be influenced by various lifestyle factors, and managing the nutritional status is the biggest key preventive measure, particularly for aged population. Also, a recent article by Laird et al. (Laird et al., 2017) reports that high yoghurt intake is associated with increased BMD and physical functions in older adults. This study further supports and strengthens our findings that administration of LA (common bacterial strain in yoghurt) enhances bone health. Thus, supplementation of our diet with LA (a common component of our diet) can be the best preventive strategy for handling osteoporosis. In summary, these results have direct clinical implications in the management and prevention of post-menopausal osteoporosis which affects every 4^th^ women and every 5^th^ men in the world (Haczynski and Jakimiuk, 2001).

## Appendix A Supplementary data

Supplementary figures Image 1
