# Supplementary material for: Lactobacillus acidophilus inhibits bone loss and increases bone heterogeneity in osteoporotic mice via modulating Treg-Th17 cell balance
Source: Bone Rep. 2018 Feb 5;8:46–56. doi: 10.1016/j.bonr.2018.02.001 (PMC6019967; doi:10.1016/j.bonr.2018.02.001)
Supplement: Supplementary file 1 — Supplementary figures [file mmc1.pptx]

## Slide 1
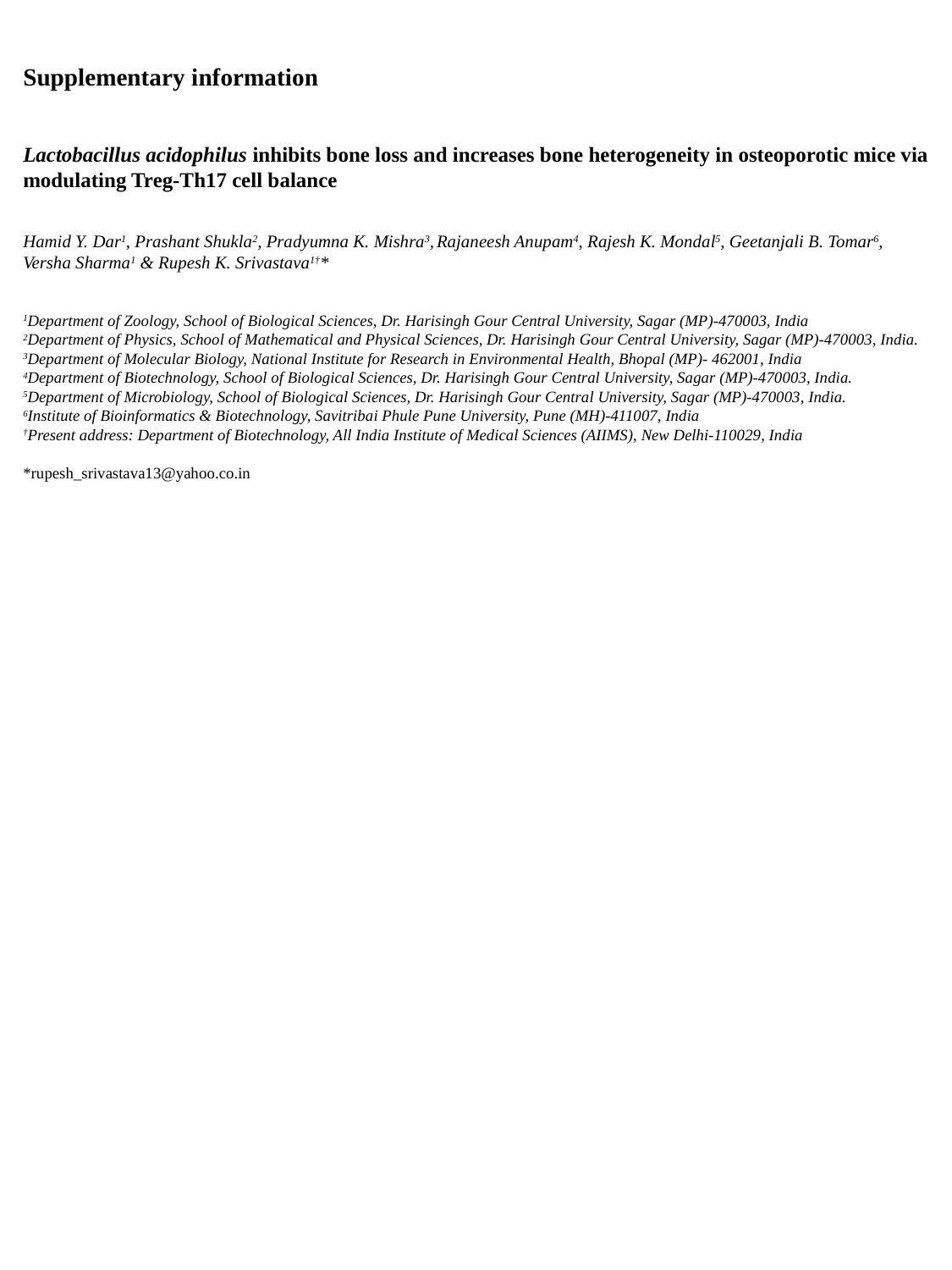

Supplementary information
Lactobacillus acidophilus inhibits bone loss and increases bone heterogeneity in osteoporotic mice via modulating Treg-Th17 cell balance
Hamid Y. Dar1, Prashant Shukla2, Pradyumna K. Mishra3, Rajaneesh Anupam4, Rajesh K. Mondal5, Geetanjali B. Tomar6, Versha Sharma1 & Rupesh K. Srivastava1†*
1Department of Zoology, School of Biological Sciences, Dr. Harisingh Gour Central University, Sagar (MP)-470003, India
2Department of Physics, School of Mathematical and Physical Sciences, Dr. Harisingh Gour Central University, Sagar (MP)-470003, India.
3Department of Molecular Biology, National Institute for Research in Environmental Health, Bhopal (MP)- 462001, India
4Department of Biotechnology, School of Biological Sciences, Dr. Harisingh Gour Central University, Sagar (MP)-470003, India.
5Department of Microbiology, School of Biological Sciences, Dr. Harisingh Gour Central University, Sagar (MP)-470003, India.
6Institute of Bioinformatics & Biotechnology, Savitribai Phule Pune University, Pune (MH)-411007, India
†Present address: Department of Biotechnology, All India Institute of Medical Sciences (AIIMS), New Delhi-110029, India
*rupesh_srivastava13@yahoo.co.in

## Slide 2
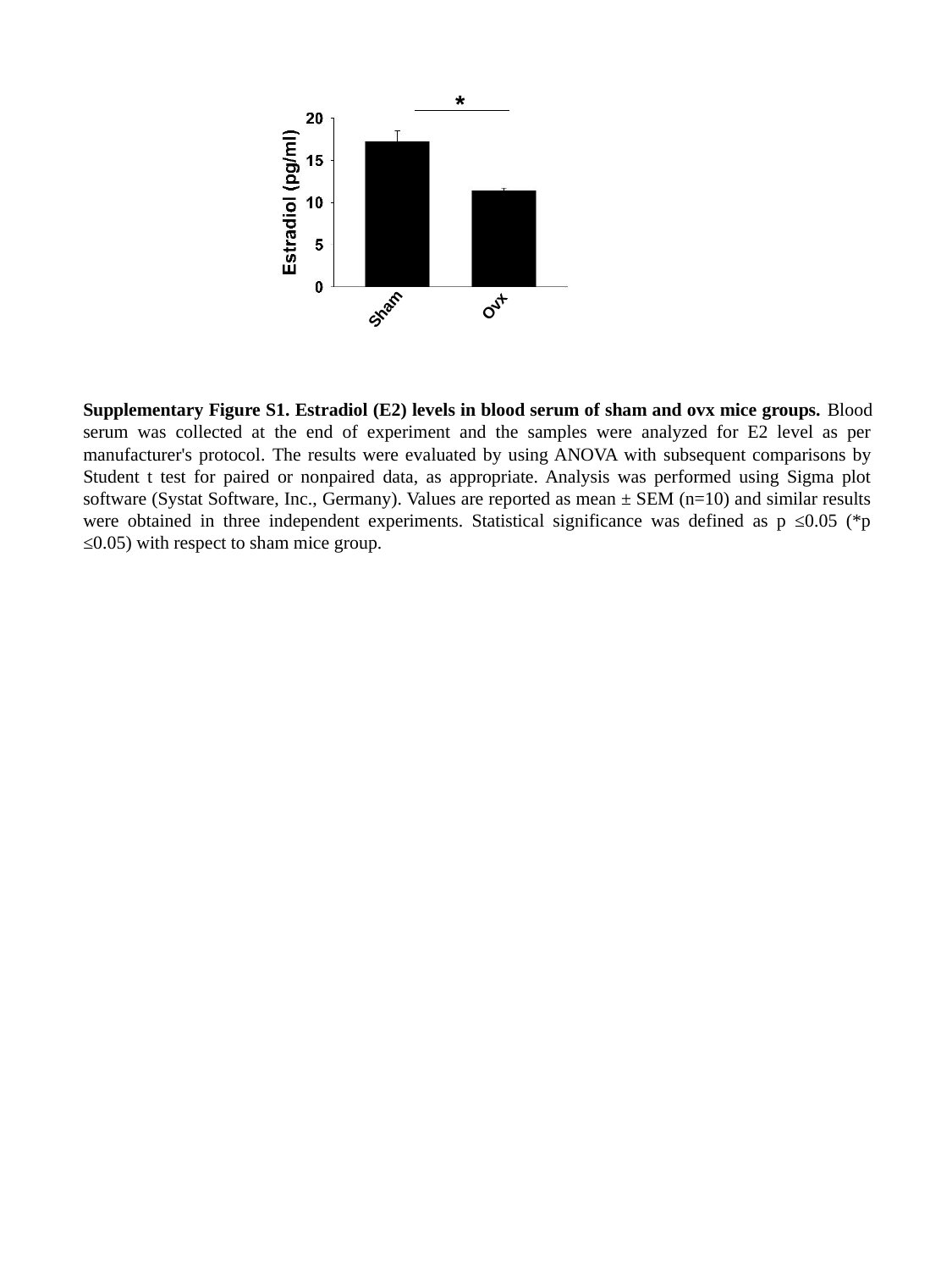

*
Ovx
Sham
Supplementary Figure S1. Estradiol (E2) levels in blood serum of sham and ovx mice groups. Blood serum was collected at the end of experiment and the samples were analyzed for E2 level as per manufacturer's protocol. The results were evaluated by using ANOVA with subsequent comparisons by Student t test for paired or nonpaired data, as appropriate. Analysis was performed using Sigma plot software (Systat Software, Inc., Germany). Values are reported as mean ± SEM (n=10) and similar results were obtained in three independent experiments. Statistical significance was defined as p ≤0.05 (*p ≤0.05) with respect to sham mice group.

## Slide 3
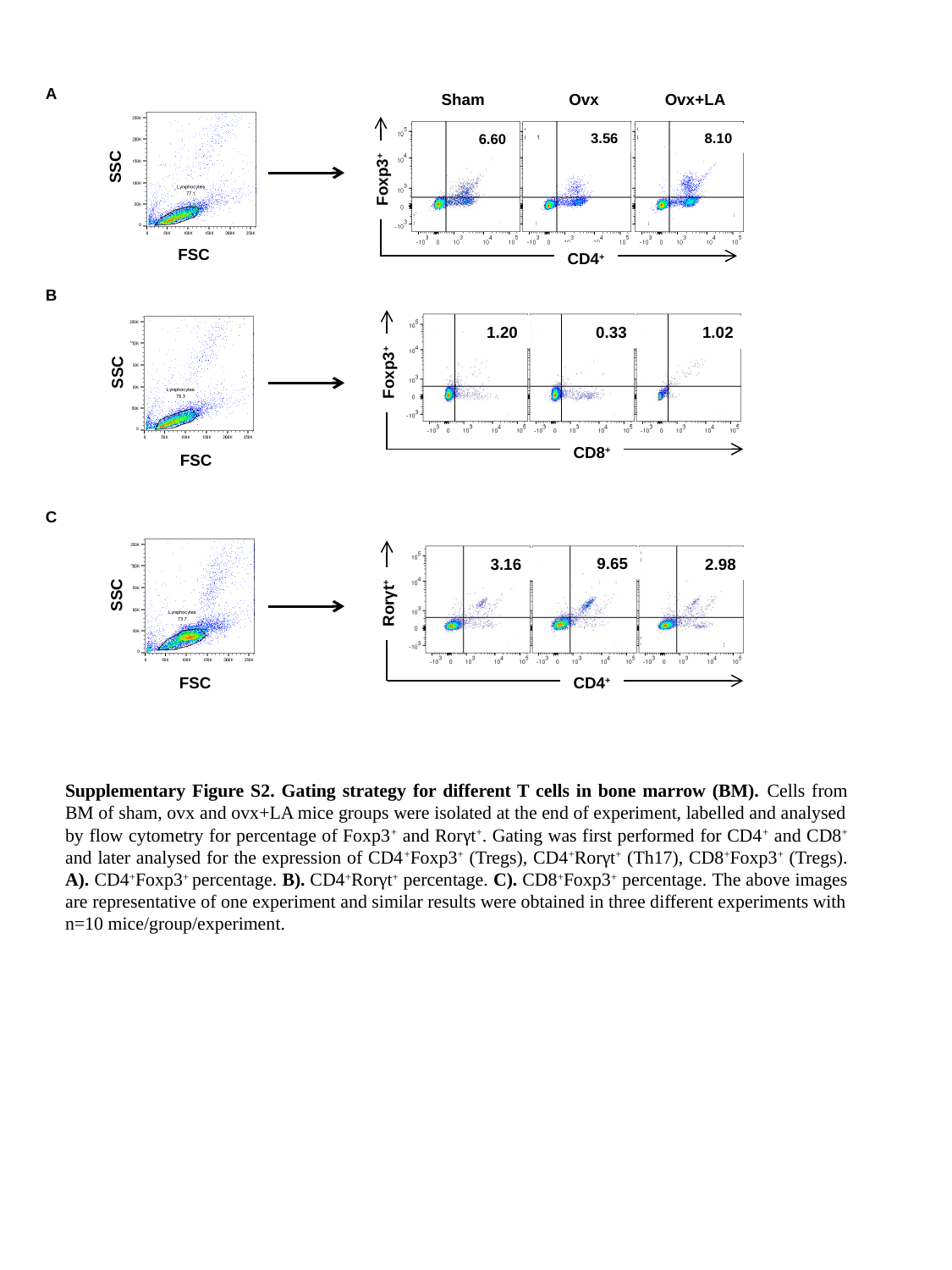

A
Sham
Ovx
Ovx+LA
SSC
FSC
6.60
3.56
8.10
Foxp3+
CD4+
B
1.20
0.33
1.02
Foxp3+
CD8+
SSC
FSC
C
SSC
FSC
3.16
9.65
2.98
Rorγt+
CD4+
Supplementary Figure S2. Gating strategy for different T cells in bone marrow (BM). Cells from BM of sham, ovx and ovx+LA mice groups were isolated at the end of experiment, labelled and analysed by flow cytometry for percentage of Foxp3+ and Rorγt+. Gating was first performed for CD4+ and CD8+ and later analysed for the expression of CD4+Foxp3+ (Tregs), CD4+Rorγt+ (Th17), CD8+Foxp3+ (Tregs). A). CD4+Foxp3+ percentage. B). CD4+Rorγt+ percentage. C). CD8+Foxp3+ percentage. The above images are representative of one experiment and similar results were obtained in three different experiments with n=10 mice/group/experiment.

## Slide 4
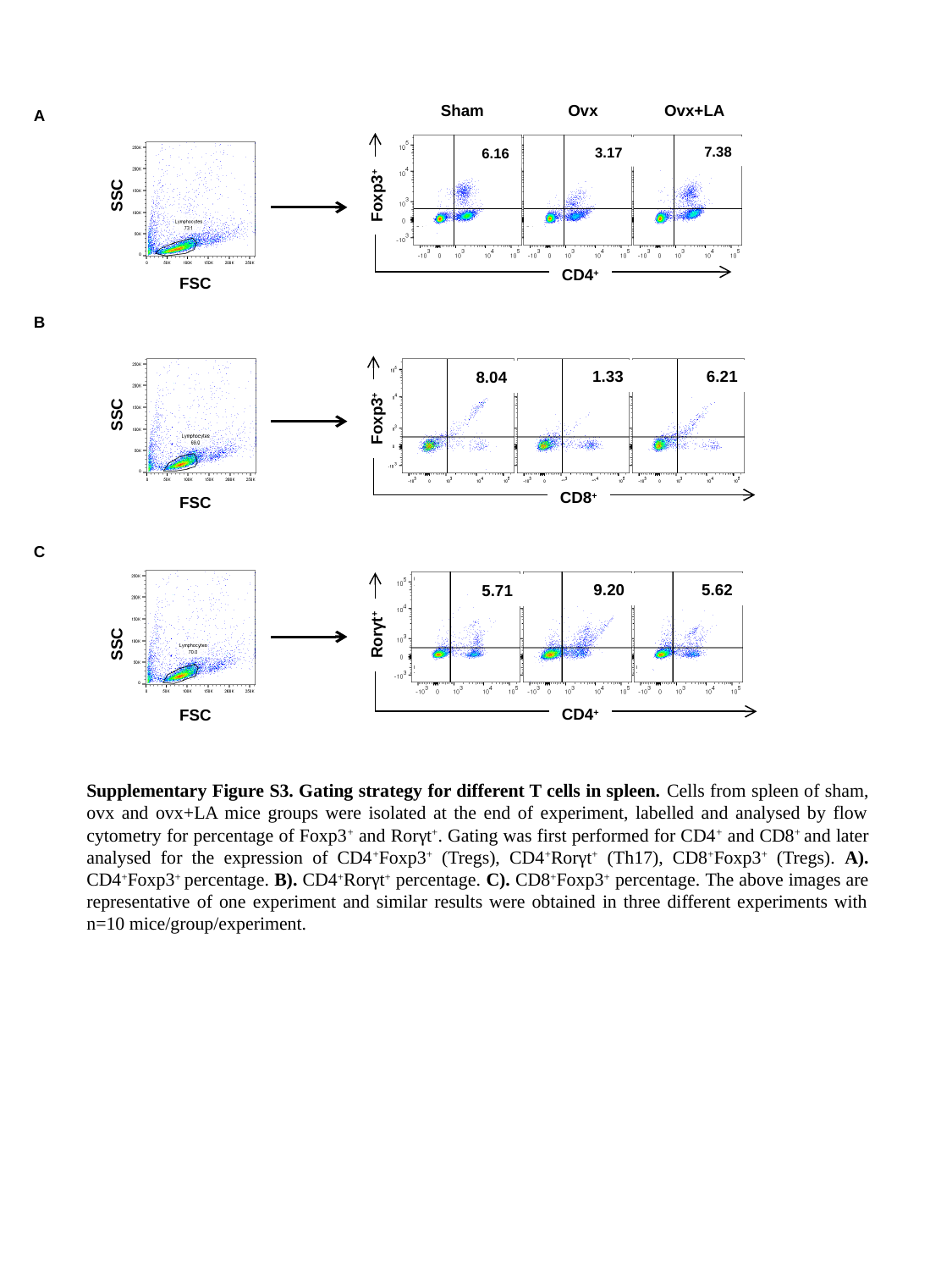

Sham
Ovx
Ovx+LA
A
6.16
3.17
7.38
Foxp3+
CD4+
SSC
FSC
B
8.04
1.33
6.21
Foxp3+
CD8+
SSC
FSC
C
5.71
9.20
5.62
Rorγt+
CD4+
SSC
FSC
Supplementary Figure S3. Gating strategy for different T cells in spleen. Cells from spleen of sham, ovx and ovx+LA mice groups were isolated at the end of experiment, labelled and analysed by flow cytometry for percentage of Foxp3+ and Rorγt+. Gating was first performed for CD4+ and CD8+ and later analysed for the expression of CD4+Foxp3+ (Tregs), CD4+Rorγt+ (Th17), CD8+Foxp3+ (Tregs). A). CD4+Foxp3+ percentage. B). CD4+Rorγt+ percentage. C). CD8+Foxp3+ percentage. The above images are representative of one experiment and similar results were obtained in three different experiments with n=10 mice/group/experiment.
